# Sialendoscopy approach in treating juvenile recurrent parotitis: a systematic review

**DOI:** 10.1186/s40463-023-00658-1

**Published:** 2023-08-19

**Authors:** D. Soriano-Martín, L. García-Consuegra, L. Junquera, T. Rodríguez-Santamarta, S. Olay, S. Junquera-Olay

**Affiliations:** 1 Department Maxillofacial Surgery, Central University Hospital of Asturias, Avenida de Roma, 33011 Oviedo, Spain; 2 Department of Surgery, Oviedo University, Julian Clavería, 33009 Oviedo, Spain; 3 Department of Radiology, San Agustin University Hospital, 33410 Heros Avilés, Spain

**Keywords:** Sialendoscopy, Juvenile recurrent parotitis, Sialadenitis, Salivary gland

## Abstract

**Background:**

Juvenile recurrent parotitis (JRP) is characterized by recurrent episodes of painful parotid swelling in children. The purpose of this systematic review was to determine the diagnostic and therapeutic effectiveness of sialendoscopy in children affected by JRP.

**Methods:**

A systematic literature search was performed in PubMed, EMBASE, Scopus and the Cochrane Library until April 2022, without language restrictions or specified start date. Quality assessment was performed using the Newcastle–Ottawa Scale (NOS).

**Results:**

Our review included 524 patients and 646 sialendoscopies. The sample sizes of the different studies ranged from 3 to 77 subjects. Most authors performed sialendoscopy under general anesthesia. The mean percentage of recurrences observed was 25.1% (95% confidence intervals) (CI 23.6–26.6). There was a statistically significant relationship between the number of attacks/year and recurrences (*p* < 0.05). The percentage of recurrences according to the type of irrigation/flushing used ranged from 22.2% to 25.2%, with no significant differences between the use of corticosteroids alone (25.2% of recurrences), corticosteroids plus antibiotics (25% of recurrences) or saline alone (22.2% of recurrences). Sialoendoscopy has proved in all cases to be a valid method for the diagnosis of JRP, but it does not allow a reliable differential diagnosis with other autoimmune parotitis such as Sjögren's syndrome.

**Conclusion:**

According to our results, parotid sialoendoscopy was 74.9% effective as a primary treatment in the prevention of recurrent symptoms in JRP. The type of ductal irrigation used did not significantly influence the prognostic outcome.

**Supplementary Information:**

The online version contains supplementary material available at 10.1186/s40463-023-00658-1.

## Introduction

Juvenile recurrent parotitis (JRP) is a nonspecific sialadenitis with recurrent inflammation of parotid glands in children. Sialadenitis in the pediatric population accounts for up to 10% of all salivary gland disease. JRP is the second most common cause of parotitis in childhood, only after paramyxovirus (the mumps). Other potential etiologies of parotitis include: bacterial infection, autoimmune disorders, including Sjögren's syndrome and lupus [1, 2].

Clinical symptoms of JRP include recurrent parotid swelling and/or pain, associated with fever and malaise. JRP is commonly unilateral, but can occur bilaterally with symptoms usually more prominent on one side. The age of onset is bimodal, with a peak incidence at around 3 to 6 years and around 9 to 11 years of age. In the majority of patients symptoms resolve at adolescence [3, 4].

Recently Garavello et al. [5] suggested the following inclusion criteria: age < 16 years, recurrent unilateral or bilateral painful parotid swelling and at least 2 episodes during the last 6 months, as well as the following exclusion criteria: obstructive lesions, dental malocclusion, Sjögren syndrome, and IgA deficiency.

JRP diagnosis is based on the clinical picture and can be confirmed by ultrasonographic study. At this diagnostic procedure, typical findings are distal small roundish hypoechoic areas in the glandular parenchyma, corresponding to ductal dilatation, duct lymphocytic peripheral infiltration, or enlarged intraparenchymal lymph nodes [6]. Some studies also describe the use of magnetic resonance (MR) and MR sialography for diagnosis of JRP [7, 8].

Since 2004 different authors [9] have evaluated sialendoscopy for the diagnostic and therapeutic management of JRP. High success rates and low morbidity seem to justify the increasing use of sialendoscopy in JRP, although a comprehensive analysis of documented results has not yet been reported.

In the current study, we conducted a systematic literature review to evaluate treatment options that have emerged over the past 17 years for patients with JRP, especially focusing on the therapeutic value of sialoendoscopy. With this work we aim to answer the following two clinical questions: firstly, according to the current evidence, is sialoendoscopy the best type of treatment for patients with JRP? And secondly, does sialoendoscopy allow a differential diagnosis between JRP and other childhood autoimmune parotitis, such as Sjogren's syndrome?

## Materials and methods

### Systematic review of the literature protocol

This study was conducted according to Preferred Reporting Items for Systematic Reviews and Meta-Analysis (PRISMA) guidelines [10].

### Search strategy and study selection

The systematic literature search was performed in PubMed, EMBASE, Scopus and Cochrane Library until April 2022, without language restrictions or specified start date. The following combinations of keywords and medical subject headings were used: *sialendoscopy OR recurrent parotitis, juvenile recurrent parotitis, parotitis childhood, paediatric sialoendoscopy*. All studies were screened according to title and abstract, and eligible manuscripts were retrieved for full-text review. In addition, the reference lists of each original and review article were hand searched to avoid omitting potential studies. The literature search was conducted independently by two investigators (DS, LJ), and any disagreements were resolved by consensus. Studies selected by the search strategy and other references were managed with the RefWorks program, and duplicate articles were removed with the associated tools.

### Eligibility criteria

Studies that met the following criteria were included: (a) case reports, case series, prospective clinical trials, nonrandomized and randomized studies, and observational studies; (b) the main focus of the article describes more than one sialendoscopy procedures in the treatment of JRP; (c) the study includes children (< 18 years) with at least two or more episodes of intermittent swelling of the parotid glands on one or both sides during the past 6 months; and (d) the study mentions the gland(s) affected, clinical criteria to sialendoscopy procedures, the endoscopy findings, type of anesthesia, irrigation/lavage method, recurrence, a follow-up period and postoperative complications. The exclusion criteria were as follows: (a) the number of sialoendoscopies in JRP or the number of patients with recurrence after the first sialoendoscopy in the study were not clearly identified; (b) studies including patients with sialolithiasis, dental malocclusion, Sjogren syndrome, congenital IgA immunodeficiency, and relevant systemic diseases; (c) studies that included adult patients (age > 18 years), with the exception of the article by Shacham et al. [11], which dealt mainly with the pediatric population (70 patients), but also mentioned 5 adults who were included in our revision. Letters, comments and abstracts were not eligible for evaluation.

### Protocol and registration

Two investigators (DS and LJ) independently evaluated each eligible manuscript, collected the data using a prespecified form, and collated them in a Microsoft Excel spreadsheet (MicrosoftCorp. Redmond, WA, USA). Any disagreement between reviewers was resolved by consensus. The following information was collected from each study: author, year of publication, number of patients, number of sialendoscopies, age, gender, number of clinical events per month for diagnosis, recurrences after treatment, follow-up time expressed in months, main sialendoscopic findings, type of irrigation-washing used, type of anesthesia, use of prophylaxis or associated antibiotic treatment, and presence of complications after the procedure.

### Assessment of risk bias

Three independent investigators (DS, LJ, and SJ) used the Newcastle–Ottawa Scale (NOS) [12] to assess the individual quality of selected studies, and discrepancies were resolved by consensus. The NOS assesses the quality of nonrandomized studies based on design, content, and ease of use aimed at the task of incorporating quality assessments into the interpretation of meta-analytic results. This "star system" assigns up to a maximum of nine points for the least risk of bias in three domains: (a) selection of study groups (four points), (b) comparability of groups (two points), and (c) ascertainment of exposure (three points) for case–control and cohort studies, respectively. The NOS score ranged from 0 to 9 stars and the validity criteria were as follows: 8–9, high quality; 6–7, medium quality; < 5 low quality.

Levels of evidence were assigned according to the Oxford Centre for Evidence based Medicine [13].

### Statistical analysis

A pooled analysis of the selected studies was performed, weighted by the number of patients in each one. Comparisons on demographic and clinical categorical variables were performed using chi-square tests. Comparisons between groups on continuous measures were conducted using 2-sample independent t-tests and Anova test. A two-tailed P value of less than 0.05 was considered statistically significant. All statistical calculations were performed using SPSS software 27.0.1.

## Results

### Study selection

**Fig. 1:**
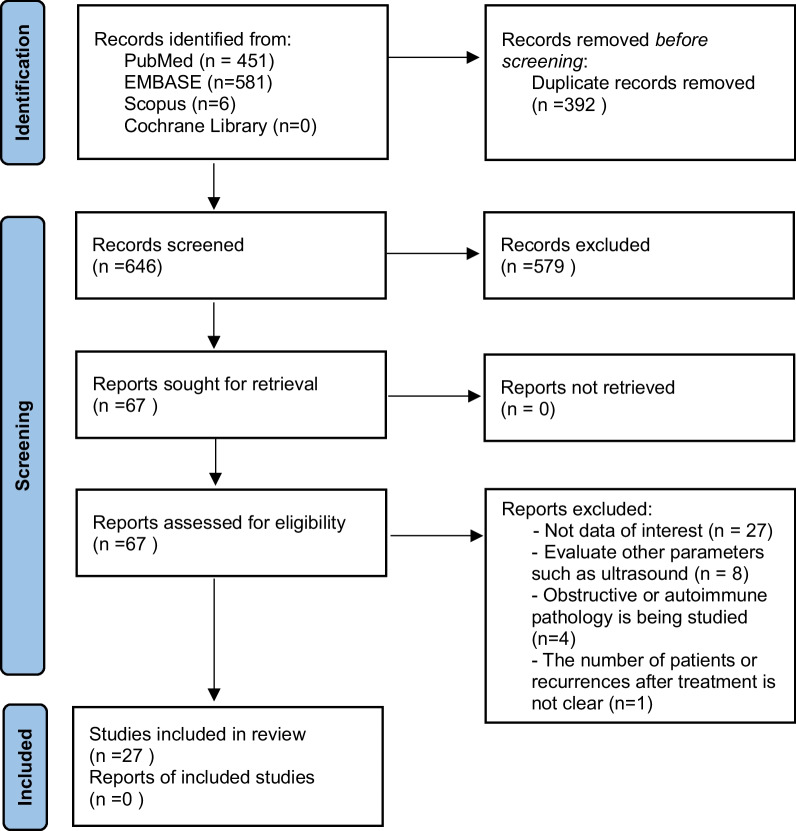
PRISMA flow diagram

A total of 1038 articles were identified in the reviewed electronic databases. The PRISMA flow diagram of the identified studies is shown in Fig. 1. We ultimately included 27 studies. They were all published during the last seventeen years**.**

### Study characteristics

The individual characteristics of the 27 included studies analyzing the value of sialendoscopy in the treatment of JRP are summarized in Tables 1 and 2. Only retrospective observational studies were found, mostly case series and three systematic reviews. The sample sizes of the different studies ranged from 3 to 77 subjects. Our review included 524 patients and 646 sialendoscopies. No randomized clinical trials are documented. There are no true meta-analyses. The age range was 1 to 18 years. The mean age of patients undergoing sialendoscopy obtained from 23 of the reviewed studies was 7.7 years. Although not statistically significant, sialoendoscopy is performed more frequently in boys (257 cases) than in girls (178 cases). Different authors do not document the gender variable in their work (89 cases) [14–20].

**Table 1:** Variables analyzed

Author	N. of cases	N. of procedures	Age (range)	Gender	Clinical criteria (swelling events/months)	Recurrence	Follow-up range (months)
Benaim et al. [31]	17	17	7,2 (11,4–3)	M 7	> 3	52,9%-24%	N/A
Borner et al. [14]	4	7	N/A	N/A	N/A	2 (50%)	> 6
Velasquez et al. [27]	18	18	< 18	M 10	> 2/12 m	8(44,4%)	22
Capaccio et al. [35]	6	8	10,2 (8–13)	M 3	≥ 2	0 (0%)	12 (11–13)
Iordanis et al. [21]	77	77	9,6 (3,5–12)	M 47	≥ 2/6 m	14 (18%)	28,8 (24–47)
Kanerva et al. [15]	20	N/A	10 (3–16)	N/A	N/A	2 (10%)	70 (6–132)
Gellrich et al. [16]	15	N/A	NA	N/A	≥ 5/12 m	8 (53%)	36 (9–12)
Nation et al. [17]	19	N/A	9,65 (≤ 18)	N/A	≥ 2	5 (26%)	12–72
Berlucchi et al. [28]	23	34	7 (4–12)	M 12	≥ 2/12 m	15 (65,2%)	30 (6–70)
Faizal et al. [22]	22	29	10.7 (3–18)	M 14	≥ 2/6 m	10(45%)	6–36
Capaccio et al. [34]	32	42	7.2 (1–16)	F 19	≥ 2	7(22%)	23 (6–55)
Singh et al. [36]	17	26	5.6 (3–11)	F 9	≥ 6/12 m	7 (41,1%)	6–36
Honnet et al. [29]	5	6	6.8 (5–11)	M 4	≥ 2/12 m	0(0%)	9–29
Su et al. [33]	4	4	3.7(2–8)	M 3	≥ 2	0(0%)	≥ 3
Papadopoulou et al. [23]	12	12	8,2(4–16)	M 7	≥ 2/6 m	4 (33%)	12–48
Semensohn et al. [18]	9	13	9(3–18)	N/A	≥ 2/12 m	2 (22%)	16,5 (1–49)
Mikolajczak et al. [32]	9	10	7.1(3–13)	M 6	≥ 2	1 (11%)	15 (8–26)
Ardekian et al. [7]	50	57	N/A (2–16)	M 33	≥ 2/12 m	7(14%)	24 (12–48)
Schneider et al. [19]	15	21	6(2–15)	N/A	≥ 2	9 (60%)	12
Hackett et al. [30]	12	19	9,7 (6–16)	M 7	≥ 2/12 m	4 (33%)	1–24
Gary et al. [24]	3	3	9 (6–11)	M3	≥ 2/6 m	0 (0%)	3–16
Konstantinidis et al. [25]	6	7	9,4 (8–11)	M 3	≥ 2/6 m	2 (33%)	14 (12–17)
Jabbour et al. [1]	5	8	6.5 (3–8)	M 5	≥ 2	3 (60%)	22 (7–33)
Martins-Carvalho et al. [20]	18	18	N/A	N/A	N/A	4 (22%)	24 (4–24)
Shacham et al. [11]	70	93	6,7 (1–40)	M 43	≥ 2/12 m	14 (20%)	6–36
Quenin et al. [26]	10	17	5 (1,8–13)	F 6	≥ 2/6 m	1(10%)	11 (2–24)
Nahlieli et al. [9]	26	46	7 (2,5–13)	M 14	≥ 2/12 m	2 (8%)	4–36

N/A: not available. M: male. F: female

**Table 2:** Variables analyzed (continuation)

Author	Main findings	Irrigation	Mode of anesthesia	Prophylactic antibiotics	Complications (cases)
Benaim et al. [31]	None	None. Normal saline. Steroid	GA	Clindamycin. Amoxicillin-clavulanic. Cephalosporin	N/A
Borner et al. [14]	Fibrous, stenosis	Methylprednisolone	LA /GA	N/A	N/A
Velasquez et al. [27]	Fibrinous debris, duct stenosis	Triancinolona	GA	N/A	N/A
Capaccio et al. [35]	Mucous plugs, stenosis, debris, pale duct	SS + dexamethasone	Sedation	Amoxicillin	Swelling (1 case) and pain (N/A)
Iordanis et al. [21]	N/A	SS + prednisone	GA	Amoxi-clavulanate (1 day preOP)	N/A
Kanerva et al. [15]	N/A	SS ± hydrocortisone	GA/LA	None	Swelling (1 case)
Gellrich et al. [16]	N/A	SS + prednisone	N/A	N/A	N/A
Nation et al. [17]	Sludg, stenosis	SS ± steroids (solumedrol, hydrocortisone, triamcinolone, decadron)	GA	N/A	None
Berlucchi et al. [28]	Mucous plugs, stenosis, debris, pale duct	SS + hydrocortisone	GA	Yes (postOP)	None
Faizal et al. [22]	Pale duct, stenosis	SS + hydrocortisone ± gentamicin	GA	Cefuroxime IV (preOP)	None
Capaccio et al. [34]	Mucous plugs, stenosis, pale duct	SS + betametasona	GA	Amoxi-clavulanate IV (IntraOP)	N/A
Singh et al. [36]	Mucous plugs, stenosis, debris, pale duct	SS + hydrocortisone	GA	amoxicillin– clavulanic acid IV (postOP)	None
Honnet et al. [29]	Mucous plugs, stenosis, pale duct	SS + steroid	GA	None	None
Su et al. [33]	Stenosis, debris, pale duct	SS + methylprednisolone	GA	Yes (postOP)	N/A
Papadopoulou et al. [23]	Mucous plugs, stenosis, debris, pale duct	SS + prednisone	GA/LA	N/A	None
Semensohn et al. [18]	Stenosis, debris	SS	GA	None	None
Mikolajczak et al. [32]	Pale duct, debris	SS + hydrocortisone	GA	Amoxi-clavulanate (7 days)	None
Ardekian et al. [7]	Pale duct, stenosis, floating fibers, mucous plugs	SS + steroids + penicillin	LA + sedation	Amoxicillin (7 days)	Duct perforation (3 cases)
Schneider et al. [19]	N/A	SS + prednisone	GA	Amoxi-clavulanate (5 days peri- and postOP)	N/A
Hackett et al. [30]	Stenosis, debris, pale duct	SS + steroids ± antibiotic	GA	None	Swelling and pain (2 cases)
Gary et al. [24]	Stenosis, debris, pale duct	SS + triamcinolone	GA	None	Duct stenosis (2 cases)
Konstantinidis et al. [25]	Stenosis, pale duct, fibrinous debris, purulent debris	SS + prednisone	GA/LA	Amoxi-clavulanate(pre- and postOP 48 h)	Swelling and pain (1 case)
Jabbour et al. [1]	Stenosis, debris	SS + hydrocortisone	GA	N/A	None
Martins-Carvalho et al. [20]	Pale duct, stenosis	SS + xylocaine + prednisone	GA	Amoxi-clavulanate (7 days postOP)	Duct perforation and airway obstructions (3 cases)
Shacham et al. [11]	Pale duct, stenosis	SS + hydrocortisone	GA	Amoxi-clavulanate IV postOP	Aspiration pneumonia (1 case)
Quenin et al. [26]	Pale duct, stenosis, sludge material	SS + steroids	GA	Amoxi-clavulanate (48 h postOP)	Upper airway obstruction (2 cases)
Nahlieli et al. [9]	Pale duct, stenosis, wide Stensen’s papilla	SS + hydrocortisone	GA	Amoxicillin– clavulanic acid IV (postOP)	N/A

LA: local anesthesia. GA: general anesthesia. N/A: not available. PostOP:postoperative. PreOP: preoperative. IntraOP: Intraoperative. PeriOP: perioperative period. SS: saline solution

The mean recurrence rate observed in the present systematic review was 25.1% (95% CI 23.6–26.6). The mean follow-up time was 19.4 months (95% CI 13.2–25.7). A low percentage of children (mean 14%) was submitted to a second or more sialendoscopic procedures. The indication for sialoendoscopy was the presence of two episodes of parotid swelling during the last 6 months in 130 patients [21–26]. The same number of episodes but in a 12-month period was the criterion for sialoendoscopy, which was applied in 213 patients [7, 9, 11, 18, 27–30]. In 11 studies in our review, the presence of two or more episodes of parotid swelling was reported as a criterion, but without specifying the time of presentation [1, 14, 15, 17, 19, 20, 31–35]. Two authors, Gellrich et al. [16] and Singh et al. [36] describe, in their series, the indication for the technique included patients who presented a minimum of five or six episodes of swelling in 12 months. In those patients who received sialendoscopy after presenting two episodes during the last 6 month the percentage of recurrences ranged from 21.7% to 25.8%. In the group of patients who underwent surgery after two or more episodes in 12 months, the recurrence rate ranged from 20.1% to 24.9%. With five or six episodes in the same period of time, recurrence rates ranged from 44.6% to 49%. There was a statistically significant relationship between the number of attacks/year and recurrences (p = 0.001) (Anova post hoc) (Additional file 1: Table s1).

In the present review, most of the sialendoscopies were performed under general anesthesia 80.4% (520 surgical procedures). In 7.1% were performed under general anesthesia or local anesthesia. Kanerva et al.[15] performed sialoendoscopies with local anesthesia in patients older than 10 years and Konstantinidis et al.[25] in patients older than 8 years. In 10% of the procedures were performed under sedation or sedation and local anesthesia [7, 35]. Prophylactic antibiotherapy was administered in 506 sialoendoscopies (78.3%), preferably using amoxicillin-clavulanic acid [19, 21, 25, 34]. Ductal irrigation with isotonic saline solution plus corticoids was the most commonly used modality (511 sialoendoscopies: 79.1%) [1, 9, 11, 14–17, 20, 21, 23–25, 27–29, 31–36]. a small minority of cases underwent instrumentation such as balloon dilation or microdrilling. A small minority of cases underwent instrumentation such as balloon dilation or microdrilling (136 sialoendoscopies) [1, 9, 11, 14, 20, 30]. Combined corticosteroid and antibiotic irrigation was performed in 16.3% of the sialoendoscopies (105 techniques) [7, 22, 30] and the exclusive use of saline lavage in 2% of the procedures [18, 31]. The percentage of recurrences according to the type of ductal irrigation used ranged from 22.2% to 25.2%, with no significant differences between the use of corticosteroids alone (25.2% of recurrences), corticosteroids plus antibiotics (25% of recurrences) or saline alone (22.2% of recurrences) (Additional file 1: Table s2). The most frequently sialoendoscopic finding was the presence of stenosis (95.5%), followed by pale duct (71.4%), debris (50%) and mucous plug (36.4%). However, very few authors detail the use of balloon dilatation or microdrill for treatment of stenosis [1, 9, 11, 20, 30]. The percentage of complete resolution ("cured") in the series that performed interventional sialendoscopy ranges between 92% [9] and 60% [1], without defining the specific results after the dilatation procedure.

Morbidity related to sialoendoscopy is low, being reported in 4.4% of patients. The complications described are: swelling and pain [15, 25, 30, 35], duct perforation [7, 20], proximal duct stenosis [24], rare cases of upper airway obstruction [20, 26], and the usual risks of general anesthesia [11]. In 95.6% of the patients there were no complications or this data was not available [1, 17, 18, 22, 23, 28, 29, 32, 36].

### Study quality

No randomized controlled studies were found, and all the results were based on case series. Although some authors mention the randomized work of Wen-hua et al. [37], this study does not include the evaluation of sialoendoscopy as a form of treatment.

Three systematic reviews of the literature have been documented so far. The first from 2013 includes 10 papers and 179 children [38], the second from 2015 [39] analyzes qualitative information from seven studies (120 patients), and the latest from 2018 [5] Garavello includes 24 studies analyzing 336 children. None of these reviews is a true meta-analysis. The present work includes 27 studies and analyzes variables not contemplated in the previous reviews on a sample of 524 children.

In our review, all outcomes were based on case series in the absence of a control group and randomization (level of evidence 4) [13].

The risk of bias and quality assessment was performed according to the NOS. Regarding the selection domain, most of the included studies provided an adequate description of case characteristics and selection criteria. Regarding the comparability domain, no work provided information. For the exposure domain, few studies reported on blinding of analyses or nonresponse rates. The mean NOS score in our study was 1.63. In all the selected studies the score was less than 5 and therefore of low quality. (Additional file 1: Table s3)*.*

## Discussion

JRP is defined as recurrent parotid inflammation of a non-obstructive, non-suppurative nature in a child aged 1 to 16 years. It presents as unilateral or bilateral parotid gland inflammation with 2 or more episodes occurring before puberty. It is an uncommon condition and its etiology, which is likely to be multifactorial, is unknown. Historically, JRP has been described in association with Sjögren's syndrome, hypogammaglobulinemia, IgG3 deficiency, IgA deficiency and as a frequent manifestation in patients with HIV infection. At present, JRP can be considered a sentinel sign of other diseases of immunologic/autoimmune etiology whose early diagnosis, follow-up and treatment can improve prognosis [5, 40, 41].

Originally, JRP was attributed to congenital dilatations and malformations and/or recurrent infections [42], but nowadays a multifactorial approach to etiology is more accepted. Genetically, JRP presents an autosomal dominant pattern with incomplete penetrance and variable expressions [43]. The higher concentrations of *Streptococcus pneumoniae* and *Haemophilus influenzae* isolated in the saliva of JRP patients may support an infectious etiology [31, 44]. Currently, the main cause postulated to explain its pathogenesis is decreased salivary production with insufficient salivary flow through the ductal system, which favors ascending infections of the salivary glands through the oral cavity [45].

The diagnosis of JRP is based on the clinical picture and can be confirmed by ultrasonography. In this diagnostic procedure, typical findings are: enlarged and heterogeneous parotid gland, with unilateral or bilateral involvement, and hypoechogenic areas of 2–4 mm and/or hyperemia on Doppler ultrasound indicative of sialectasia or lymphocytic infiltration [46]. The noninvasive nature of ultrasound makes it an ideal imaging modality for children. Expected ultrasound findings in JRP include scattered hypoechoic foci (referred to as "Swiss cheese" or "moth-eaten") [31].

Our demographic results are consistent with previous reports of patients with JRP. JRP most frequently affected male children. The ages of JRP onset were bimodally distributed, with a primary peak between the ages of 4 and 8 years [31, 39]. Although the criteria for JRP diagnosis postulated by Garavello et al. [5] included patients younger than 16 years, most of the reviewed papers included patients younger than 18 years.

The main criteria to establish the severity of the disease are the frequency of recurrences, the duration of the event, the severity of inflammatory symptoms and the importance of glandular alterations [3]. None of the papers in the present review specifically contemplates the influence of these variables on the results of sialoendoscopic treatment. In our review and based on the number of episodes of parotid swelling before sialoendoscopy, we observed the existence of a statistical relationship between the number of episodes/year and recurrences. A higher number of swelling episodes in a shorter time presents a higher probability of recurrence after sialoendoscopy. However, the specific number of episodes from which the different authors proceed to perform sialoendoscopy is not clearly defined in many publications. The frequency of these acute episodes is variable and ranges from 2 to > 10 per year. Studies with a more specific design would be necessary to analyze the possible influence of the number of episodes of inflammation and the efficacy of sialoendoscopy. However, in an exploratory manner, the present review points to a possible relationship.

The mean percentage of recurrences observed in our systematic review was 25.1% (95% CI 23.6%-26.6%). This result is similar to that reported in previous studies. In 179 children included in 10 studies, Canzi et al. [38] observed complete evanescence of symptoms after sialendoscopic treatment in 78% of patients and partial regression in 22% of cases. In the review by Ramakrishna et al. [39], based on 7 studies with 120 patients and 165 glands, the primary success rate for interventional sialoendoscopy was 73% (95% CI 64–82). The review by Garavello et al. [5] of 336 children showed that only 25.8% (95% CI 21.5–30.8) of treated children had further recurrences. Nevertheless, there is little information on the number of sialoendoscopies that should be performed to achieve clinical resolution of the pathology. Some authors reported that even one sialendoscopic session may be sufficient to cure the patient [11, 15, 36], while others observed an improvement, and not a cure. In this regard, the serie of 17 patients by Benaim et al. [31], provides interesting information. In their study, the success rate after the first sialoendoscopy was 47.1%, after the second sialoendoscopy it was 17.6% and after the third it was 11.8%. In short, for these authors, an overall success rate of 76.5% was only achieved after three sialoendoscopies. Generically, more than one sialoendoscopy would be necessary to obtain complete resolution ("cured"). The mean value of repeat procedures observed in the present study was 14%, with a range of 0% [24] al 25% [30].

In the present study, the most frequently sialoendoscopic finding was the presence of stenosis, followed by pale ductus, debris and mucous plug. These results are not in agreement with previous studies. In the study by Canzy et al. [38] the most relevant and recognized sialoendoscopic finding was the white appearance of the wall and the lack of vascularity in the ductal layer (mean 75%). Confined or diffuse stenosis and multiple fibrinous debris/mucous plugs were observed in a high percentage of children (mean 56% and 45%, respectively) [7, 9, 25, 38]. Nevertheless, the percentage of dilatations reported by different authors during sialoendoscopy was lower than the recognized percentage of stenosis [1, 9, 11, 30].

Histologically in patients with JRP there are intraductal cystic dilatations of peripheral ducts with periductal lymphocytic infiltration, called as sialectasis. The ecstatic ducts are usually 1-2 mm in diameter and typical have a white appearance of the ductal layer without the healthy blood vessel coverage, when compared with a normal gland [9, 45].

Although an international consensus on the classification of parotid duct stenosis has not yet been achieved, recent publications suggest that stenoses can be classified into up to three groups: inflammatory (type 1); fibrous, associated with circular or web-like intraductal inclusions, often with only a moderate degree of luminal narrowing (type 2); and fibrous, affecting the entire ductal wall, almost always with high-grade to complete obstruction (type 3) [47]. This diagnostic information could be reached indirectly with different imaging methods such as ultrasound or sialography. In adult patients, sialendoscopy is considered to be performed in 33.3% of type 1 stenoses, 52.9% of type 2 stenoses, and 77.1% of type 3 stenoses. Cortisone lavage guided by sialendoscopy was sufficient in 73% of cases of type 1 stenosis. Interventional sialendoscopy with instrumental dilation was successful in more than 47.1% of cases of type 2 and 3 stenosis [48].

In children, at present, ultrasound would be the best imaging option for suspected inflammatory ductal pathology or ductal stenosis; it can provide a diagnosis in the parotid gland in most cases and could contribute to the indication for sialendoscopy and its control efficacy. Recently Goncalves et al. [49] observed that parotid glands with normal sialendoscopic findings had a duct diameter of 0.3 mm (0–2.7 mm) and homogeneous hyperechoic parenchyma on ultrasound in 98.7%. Ductal inflammation/sialodochitis on sialendoscopy had significantly larger ductal diameter of 0.7 mm (0–4.3 mm) and hypoechoic parenchyma in 78.%. Parotid glands with stenosis had hypoechoic parenchyma in 52.6% and a ductal diameter of 4.1 mm (0–19.0 mm). The ductal diameter was ≥ 2.7 mm in 95.6% of stenoses. Nonetheless, to our knowledge, there are no studies using preoperative and postoperative ultrasound to compare the efficacy of sialoendoscopy.

Treatment of JRP in the acute phase is based on a combination of sialogogues, parotid gland massage and antibiotics [5]. In the serie by Schneider et al. [19], thirty-six patients were treated over a period of 79 months, 15 with salivary endoscopy with cortisone irrigation and 21 with antibiotic therapy alone. A significant reduction in recurrent episodes and pain intensity after therapy was observed in both groups. With respect to these two outcomes, the comparison showed two therapeutic options of equal marketability. However, patients with JRP who underwent sialendoscopy had significantly higher costs of care during the observation period compared to those who did not undergo the procedure, with no statistically significant difference in outcomes [19]. Subsequent work, also with a limited number of patients, reported similar results [50].

Interestingly, diagnostic sialography was also found to have a therapeutic effect, which has been attributed to the irrigation effect and potential antibacterial activity of the iodine-based contrast material [2, 51]. However in children, sialoendoscopy would avoid the radiation of sialography.

Different authors have evaluated the efficacy of sialendoscopy associated with lavage for the prevention of recurrence, as well as the efficacy of the various lavage solutions [52]. Lavage seems to break the vicious circle of decreased secretion, stasis, and infection by evacuating mucus plugs and intraductal debris. The optimal lavage solution and dilation site have not yet been defined. In our review, the various intraductal lavage solutions (corticosteroids, antibiotics, or saline) appear to be effective, and no one solution has been shown to be superior to another. Direct lavage through the parotid duct also appears to be effective and remains a treatment option after confirmation of the diagnosis by ultrasound and/or MR sialography [53].

It has recently been published that irrigation of the affected gland with 3–10 ml saline solution without any type of anesthesia is a reasonable, simple, and minimally invasive treatment alternative for JRP. Nonetheless, it is a retrospective study that only included 11 boys (age 3.3–11 years) [51]. For the authors of this study, the effect of mechanical manipulation by introduction and advancement of the endoscope remains unknown. Touching the walls of the inflamed duct with the relatively sharp tip of the endoscope may have no relevance, but could theoretically lead to scar formation. Canzi et al. [38] stated that possible side effects of sialendoscopy were ductal breach (up to 8%), proximal duct stenosis (up to 66%), and upper airway obstruction. In contrast, the intravenous catheter used by these authors was soft and flexible and was only introduced into the most distal part of the duct. Complications related to sialendoscopy were minor, but were reported in 4.4% of the procedures in our review. Some authors reported upper airway obstruction in 0.1% of patients due to parotid inflammation of the pharyngeal portion of the gland. In all cases, these events were self-limited and resolved spontaneously within 24 h [20, 26].

In our review all studies demonstrated the diagnostic value of sialoendoscopy by visualizing stenosis, hypovascularization and intraductal whitish debris. However, these sialoendoscopic findings do not allow to reach a differential diagnosis between JRP and other autoimmune parotitis of childhood such as Sjogren's syndrome [52].

Different authors point out that many children with JRP or persistent salivary gland enlargement of unknown etiology are likely to be diagnosed with Sjogren's syndrome (SS) after appropriate testing. Nevertheless, failure to meet the existing criteria for SS in adults does not exclude the diagnosis of SS. In the case of these children, continued observation with periodic repetition of tests (imaging, serological, functional) is crucial to assess progression to SS, but sialendoscopy has no diagnostic value [54–56].

Direct hospital costs one year before and after the sialendoscopy procedure in children were recently collected and analyzed. To estimate the cost of care, we obtained direct hospital costs per clinical encounter (pediatric otolaryngology, emergency room and primary care provider visits), imaging modality, outpatient antibiotic prescriptions, and for the sialendoscopy procedure, anesthesia, and post-anesthesia care unit costs from institutional administrative sources. Costs of the sialendoscopy and related expenses in patients with JRP including anesthesia and post-anesthesia care unit cost were $13,506. Mean total hospital costs were significantly higher in patients with JRP one year before and after the sialendoscopy ($4308.8 vs. $3330) compared to patients with sialolithiasis [27].

Previously, other authors compared the mean costs of care for patients with JRP treated with sialendoscopy compared to those treated conservatively. Mean costs were much higher in the sialendoscopy group ($31,338 per patient vs. $698 per patient), although treatment outcomes did not differ significantly [50]. However, in adults, the costs of sialendoscopy are usually lower than those derived from other more aggressive surgical techniques [57].

The limitations of this study include results based on case series in the absence of a control group and randomization (Level of evidence: 4). Although the number of procedures reviewed was high (646 sialendoscopies), different variables were not collected or were not uniform across the different series consulted. Due to the recurrent nature of JRP, it is possible that some patients experience a recurrence of symptoms and have not yet followed up. In addition, the different studies were often performed by the same teams, which may be a source of bias. In some studies, sialography was performed by sialendoscopy, which could lead to overestimation of the efficacy of sialendoscopy. The mean value of the 27 studies reviewed on the Newcastle–Ottawa scale is low.

## Conclusions

According to our results, parotid sialoendoscopy was 74.9% effective as primary treatment in the prevention of recurrent symptoms in JRP. The percentage of recurrences, depending on the type of ductal irrigation used, showed no significant differences between the use of corticosteroids alone (25.2% of recurrences), corticosteroids plus antibiotics (25% of recurrences) or saline solution alone (22.2% of recurrences). Morbidity related to the sialoendoscopy procedure was low and mild in severity. Multicenter, prospective, randomized and comparative trials are needed to determine more clearly the role of sialendoscopy.

### Supplementary Information

**Additional file 1. Table s1** One-way ANOVA and Bonferroni test. Statistical relationship between the variables recurrence and number of episodes of swelling before sialendoscopy. **Table s2** One-way ANOVA. Statistical relationship between the variables recurrence and type of ductal lavage used. **Table s3** Newcastle-Ottawa Scale (NOS).

## Data Availability

The datasets used and analyzed during the current study are available from the corresponding author on reasonable request.

## Abbreviations

JRP: Juvenile recurrent parotitis
MR: Magnetic resonance
PRISMA: Preferred reporting items for systematic reviews and meta-analysis
NOS: Newcastle–Ottawa Scale
N/A: Not available
M: Male
F: Female
LA: Local anesthesia
GA: General anesthesia
PostOP: Postoperative
PreOP: Preoperative
IntraOP: Intraoperative
PeriOP: Perioperative period
SS: Saline solution
