# Supplementary material for: Sialendoscopy approach in treating juvenile recurrent parotitis: a systematic review
Source: J Otolaryngol Head Neck Surg. 2023 Aug 19;52:53. doi: 10.1186/s40463-023-00658-1 (PMC10440031; doi:10.1186/s40463-023-00658-1)
Supplement: Supplementary file 1 — Additional file 1. Table s1 One-way ANOVA and Bonferroni test. Statistical relationship between the variables recurrence and number of episodes of swelling before sialendoscopy. Table s2 One-way ANOVA. Statistical relationship between the variables recurrence and type of ductal lavage used. Table s3 Newcastle-Ottawa Scale (NOS). [file 40463_2023_658_MOESM1_ESM.docx]

|  | ***mean*** | ***sd*** | ***95% CI*** | ***Sig.*** | ***Bonferroni*** | | | |
| --- | --- | --- | --- | --- | --- | --- | --- | --- |
|  |  |  |  |  |  |  | *Mean difference* | *Sig.* |
| ***2/6 months*** | 23.8 | 11.8 | 21.7-28.8 | 0.001 | ***>5/12 months*** | ***2/6 months*** | 23.1(16.1-29.9) | 0.001 |
| ***2/12 months*** | 22.5 | 16.9 | 20.1-24.9 |  |  | ***2/12 months*** | 24.3(17.6-30.9) | 0.001 |
| ***>5/12 months*** | 46.8 | 6.1 | 44.6-49.1 |  |  |  |  |  |

STABLE 1

|  | ***mean*** | ***sd*** | ***95% CI*** | ***Sig.*** |
| --- | --- | --- | --- | --- |
|  |  |  |  |  |
| ***Steroids+antibiotics*** | 25.0 | 13.9 | 21.9-28.0 | 0.857 |
| ***Steroids*** | 25.2 | 17.1 | 23.5-26.9 |  |
| ***Saline solution*** | 22.2 | 0 | 22.2-22.2 |  |

STABLE 2

| **Study** | **Selection** | | | | **Comparability** | | **Exposure** | | | **Total quality score** |
| --- | --- | --- | --- | --- | --- | --- | --- | --- | --- | --- |
|  | **Adequate case definition** | **Representativity of the cases** | **Selection of control subjects of exposure** | **Definition of control** | **Comparability of cases and controls on the basis of study design** | **Comparability of cases and controls on the basic of analysis** | **Ascertainment of exposure** | **Same method of ascertainment of exposure case control length** | **Non-response rate** |  |
| Benaim et al. 2022 (31) | x | x |  |  |  |  |  |  |  | 2/9 |
| Borner et al. 2022 (14) | x |  |  |  |  |  |  |  |  | 1/9 |
| Velasquez et al.   2022 (27) | x | x |  |  |  |  |  |  |  | 2/9 |
| Capaccio et al. 2021 (35) | x |  |  |  |  |  |  |  |  | 1/9 |
| Iordanis et al. 2021 (21) | x | x |  |  |  |  |  |  |  | 2/9 |
| Kanerva et al. 2020 (15) | x |  |  |  |  |  |  |  |  | 1/9 |
| Gellrich et al. 2020 (16) | x |  |  |  |  |  |  |  |  | 1/9 |
| Nation et al. 2019 (17) | x |  |  |  |  |  |  |  |  | 1/9 |
| Berlucchi et al. 2018 (28) | x | x |  |  |  |  |  |  |  | 2/9 |
| Faizal et al. 2017 (22) | x | x |  |  |  |  |  |  |  | 2/9 |
| Capaccio et al. 2017 (34) | x | x |  |  |  |  |  |  |  | 2/9 |
| Singh et al. 2017 (36) | x | x |  |  |  |  |  |  |  | 2/9 |
| Honnet et al. 2016 (29) | x |  |  |  |  |  |  |  |  | 1/9 |
| Su et al. 2016 (33) | x |  |  |  |  |  |  |  |  | 1/9 |
| Papadopoulou et al. 2015 (23) | x | x |  |  |  |  |  |  |  | 2/9 |
| Semensohn et al. 2015 (18) | x | x |  |  |  |  |  |  |  | 2/9 |
| Mikolajczak et al. 2014 (32) | x | x |  |  |  |  |  |  |  | 2/9 |
| Ardekian et al. 2014 (7) | x | x |  |  |  |  |  |  |  | 2/9 |
| Schneider et al. 2014 (19) | x | x |  |  |  |  |  |  |  | 2/9 |
| Hackett et al. 2012 (30) | x | x |  |  |  |  |  |  |  | 2/9 |
| Gary et al. 2011 (24) | x |  |  |  |  |  |  |  |  | 1/9 |
| Konstantinidis et al. 2011 (25) | x |  |  |  |  |  |  |  |  | 1/9 |
| Jabbour et al. 2010 (1) | x |  |  |  |  |  |  |  |  | 1/9 |
| Martins-Carvalho et al. 2010 (20) | x | x |  |  |  |  |  |  |  | 2/9 |
| Shacham et al. 2009 (11) | x | x |  |  |  |  |  |  |  | 2/9 |
| Quenin et al. 2008 (26) | x | x |  |  |  |  |  |  |  | 2/9 |
| Nahlieli et al. 2004 (9) | x | x |  |  |  |  |  |  |  | 2/9 |

STABLE 3 Newcastle-Ottawa Scale (NOS).
